# Medium cut-off hemodialysis in multiple myeloma-associated cast nephropathy: A single-center experience 

**DOI:** 10.5414/CNCS112077

**Published:** 2026-08-18

**Authors:** Ana Rita Ramos, Marisa Roldão, Rita Valério Alves, Rachele Escoli, Paulo Santos

**Affiliations:** Nephrology Department, Centro Hospitalar Médio Tejo – Torres Novas, Portugal

**Keywords:** myeloma cast nephropathy, multiple myeloma, acute kidney injury, free light chains, medium cut-off hemodialysis

## Abstract

Background: Myeloma cast nephropathy (CN) is a severe complication of multiple myeloma and a frequent cause of dialysis-dependent acute kidney injury (AKI). High circulating free light chains (FLC) contribute to tubular injury, and strategies aimed at accelerating their reduction have been proposed. Medium cut-off (MCO) membranes allow enhanced clearance of middle molecules, including FLC. Clinical data regarding their implementation in routine practice remain limited. The aim of this study was to describe our institutional experience following the introduction of MCO hemodialysis in patients with dialysis-dependent CN. Materials and methods: We conducted a retrospective single-center case series including consecutive patients treated between February 2024 and December 2025. Eligible patients had dialysis-dependent AKI due to presumed or biopsy-proven CN and were managed according to a predefined protocol combining anti-myeloma therapy and intensive MCO hemodialysis (Theranova 500), consisting of eight 6-hour sessions within the first 10 days when clinically feasible. Serum FLC levels were measured before each session. Results: Six patients were included. Baseline FLC levels ranged from 1,550 to 30,000 mg/L. A marked early decline in circulating FLC was observed during the intensive MCO phase, with 4 patients achieving > 80% reduction. Treatment interruptions due to clinical instability, frequently associated with infectious complications, occurred in several cases. Renal trajectories were heterogeneous, and dialysis independence was achieved in 3 patients. Conclusion: This case series describes the real-world implementation of MCO hemodialysis in dialysis-dependent CN. Although substantial early FLC reduction was observed, renal recovery varied and appeared to be influenced by multiple clinical factors.

## Introduction 

Kidney dysfunction is a common complication of multiple myeloma, present in approximately 30 – 40% of patients at the time of diagnosis, while ~ 10% present with renal failure severe enough to require dialysis. Renal impairment may also develop during the disease course, affecting up to 25 – 50% of patients during follow-up [1]. 

High circulating levels of free light chains (FLC) play a central pathogenic role, promoting intratubular precipitation and leading to myeloma cast nephropathy (CN), the predominant cause of acute kidney injury in this setting. CN is a well-established adverse prognostic factor and is consistently associated with increased early mortality and reduced overall survival. Importantly, this poor prognosis appears to be at least partially reversible, as recovery of kidney function translates into improved renal and patient survival [2, 3]. 

Accordingly, effective reduction of circulating FLC levels is a critical therapeutic objective in this population. The introduction of modern anti-myeloma agents has significantly improved disease control by suppressing FLC production and has contributed to better patient outcomes. Nevertheless, chemotherapy does not directly remove circulating FLC, and extracorporeal strategies have therefore emerged as a complementary approach [4]. 

Conventional hemodialysis effectively removes small solutes but has a limited capacity to clear larger middle molecules, including FLC, even when high-flux dialyzers are used. κ and λ FLC differ in their molecular characteristics, with κ chains having a lower molecular weight (~ 22.5 kDa), whereas λ chains often form dimers with a higher effective molecular weight (~ 45 kDa), which may influence their extracorporeal clearance. High cut-off dialyzers were developed to overcome this limitation and allow efficient free light chain removal, but their use is constrained by significant albumin loss. More recently, medium cut-off dialyzers have been introduced to enable the clearance of middle-molecular-weight solutes, including FLC, while largely preserving albumin. Despite this technological advance, evidence supporting the clinical benefit of medium cut-off hemodialysis in CN remains heterogeneous and predominantly observational, and data on individual free light chain and renal recovery trajectories are scarce [5]. 

In our center, medium cut-off hemodialysis has been systematically implemented over the past 2 years in patients with multiple myeloma-associated acute kidney injury due to CN [6, 7]. The aim of this study was to describe the institutional experience with extracorporeal FLC removal using a medium cut-off membrane in patients with dialysis-dependent multiple myeloma-associated CN, and to evaluate its impact on FLC reduction and subsequent clinical and renal recovery in combination with anti-myeloma chemotherapy. 

## Materials and methods 

### Study design 

This was a retrospective, single-center observational study conducted at the Nephrology Department of a Portuguese hospital between February 2024 and December 2025. Consecutive eligible patients treated according to a predefined institutional protocol were included. 

Patients with dialysis-dependent acute kidney injury in the setting of newly diagnosed or relapsed multiple myeloma and presumed or biopsy-proven CN (FLC > 500 mg/L without significant albuminuria) were eligible. Patients with alternative causes of acute kidney injury, pre-existing chronic dialysis dependence, or ineligibility for anti-myeloma therapy were excluded. 

### Dialysis and treatment protocol 

Hemodialysis was performed using the theranova 500 medium cut-off (MCO) dialyzer. Extracorporeal therapy with MCO membranes was initiated as early as feasible after chemotherapy initiation, typically within 24 – 48 hours and often after initial conventional low-flow hemodialysis sessions according to clinical status and local practice. The overall treatment strategy was informed by the MYRE and EuLITE studies [6, 7]. Eight sessions were delivered within the first 10 days of dialysis initiation, each lasting 6 hours (blood flow ≥ 250 mL/min, dialysate flow ≥ 300 mL/min). Anticoagulation with unfractionated heparin and ultrafiltration were individualized according to local practice. 

The VCD regimen (21-day cycles) included bortezomib 1.3 mg/m^2^ administered subcutaneously (days 1, 4, 8, 11), dexamethasone 20 mg (days 1 – 2, 4 – 5, 8 – 9, 11 – 12), and cyclophosphamide 750 mg/m^2^ administered intravenously (day 1). Extracorporeal therapy was initiated as early as feasible after chemotherapy initiation, typically within 24 – 48 hours. Chemotherapy regimens were selected according to current guidelines and individualized to patient characteristics. 

Before each dialysis session, patients underwent laboratory assessment including complete blood count, serum creatinine, urea, electrolytes, and FLC. Electrolytes were reassessed at 3 hours and at the end of treatment when clinically indicated. 

After the initial intensive phase, patients who remained dialysis-dependent were transitioned to conventional thrice-weekly hospital-based hemodialysis according to clinical status. In the absence of renal response, MCO membranes were maintained until completion of the third chemotherapy cycle. Thereafter, dialyzer selection was left to the treating physician. Renal recovery was defined as dialysis independence at 90 days from initiation of MCO hemodialysis. 

## Case reports 

**Figure 1.**
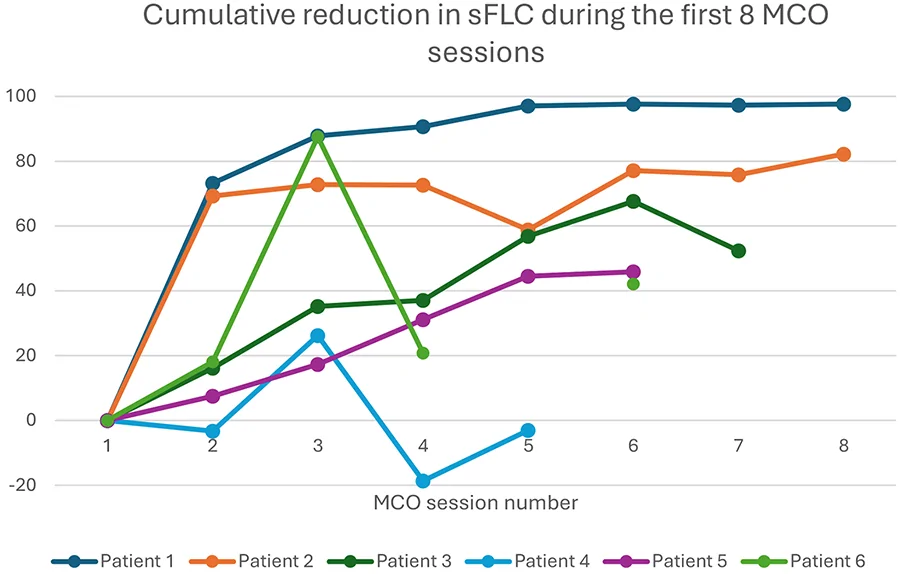
Cumulative percentage reduction in serum free light chains (FLC) during the first 8 sessions of MCO (medium cut-off) hemodialysis. Values are expressed as cumulative percentage reduction relative to the first MCO session (baseline). Not all sessions included FLC measurements.

The clinical characteristics, treatment course, and renal outcomes of all patients are summarized in Table 1. The early dynamics of cumulative serum FLC reduction during the first 8 MCO sessions are illustrated in Figure 1. 

### Patient 1 

A 77-year-old woman presented with a 3-month history of costal pain, fatigue, peripheral edema, and reduced urine output. Laboratory evaluation demonstrated normocytic anemia, severe acute kidney injury (serum creatinine 5.9 mg/dL), hypercalcemia, and radiological evidence of multiple lytic lesions. FLC was 30,000 mg/L with a markedly abnormal κ/λ ratio, consistent with newly diagnosed multiple myeloma and presumed CN. 

Renal function progressively deteriorated (serum creatinine increased to 7.6 mg/dL). Anti-myeloma therapy with VCD was initiated, and hemodialysis was started shortly thereafter for symptomatic uremia. MCO dialysis was introduced soon after chemotherapy initiation. 

The patient completed 16 MCO sessions during the initial treatment period. A substantial reduction in circulating FLC was observed, accompanied by progressive renal recovery. She was discharged dialysis-independent with serum creatinine 2.8 mg/dL. 

### Patient 2 

A 76-year-old woman presented with severe anemia, thrombocytopenia, hypercalcemia, and advanced acute kidney injury (serum creatinine 10.0 mg/dL). Further investigation revealed IgA multiple myeloma with markedly elevated FLC (20,671 mg/L), consistent with CN. Hemodialysis was initiated promptly due to symptomatic uremia and subsequently transitioned to MCO. Anti-myeloma therapy with VCD was started early during hospitalization. 

After 5 MCO sessions and completion of the first chemotherapy cycle, the clinical course was complicated by ESBL-producing *Klebsiella pneumoniae *pyelonephritis and severe pancytopenia, requiring temporary suspension of extracorporeal therapy due to clinical instability, in the absence of urgent dialysis indications during that period. Following stabilization, MCO dialysis was resumed, however, a second infectious episode with pneumonia and respiratory failure occurred, again necessitating temporary interruption of extracorporeal therapy due to clinical deterioration. 

Although a decline in circulating free light chains was documented, the planned intensive MCO protocol could not be completed as scheduled. Renal recovery was not achieved, and the patient remained dialysis-dependent at follow-up. 

### Patient 3 

A 62-year-old man presented with anemia, hypercalcemia, and acute kidney injury (serum creatinine 4.8 mg/dL). Evaluation confirmed IgG multiple myeloma with elevated FLC (1,550 mg/L). Renal function progressively worsened, requiring initiation of hemodialysis. Anti-myeloma therapy with VCD was introduced during hospitalization, and dialysis was transitioned to MCO membranes shortly thereafter. 

The patient underwent 8 MCO sessions during the initial hospitalization, with a progressive decline in circulating FLC. Hemodialysis with MCO membranes was subsequently continued 3 times weekly until completion of the third chemotherapy cycle. Despite sustained reduction in FLC levels and prolonged exposure to MCO therapy, renal recovery was not achieved, and dialysis dependence persisted. 

### Patient 4 

A 73-year-old woman presented with significant unintentional weight loss, anemia, and acute kidney injury (serum creatinine 5.0 mg/dL). During the initial hospitalization, she was diagnosed with acute pyelonephritis complicated by *Klebsiella pneumoniae* bacteremia. Progressive pancytopenia and persistent renal dysfunction prompted further investigation, which revealed markedly elevated FLC (8,660 mg/L) and a monoclonal free kappa band, consistent with light chain multiple myeloma. 

Chemotherapy with VCD was initiated on hospital day 35 after stabilization of the initial infectious episode. The patient was referred to nephrology at that time, and hemodialysis was initiated on hospital day 37 due to progressive renal deterioration. MCO dialysis was subsequently started on hospital day 42, after clinical stabilization and coordination with anti-myeloma therapy. 

During hospitalization, she completed 6 MCO sessions and received 1 full cycle and part of a second chemotherapy cycle. Hemodialysis with MCO membranes was continued 3 times weekly until completion of the third chemotherapy cycle. Despite hematologic response and sustained extracorporeal therapy, renal recovery was not achieved, and dialysis dependence persisted. 

### Patient 5 

An 82-year-old man presented with lumbar pain, anemia, hypercalcemia, and severe acute kidney injury (serum creatinine 7.5 mg/dL). FLC was markedly elevated (4,205 mg/L), consistent with newly diagnosed multiple myeloma and presumed CN. 

Renal function deteriorated during hospitalization, accompanied by declining urine output, requiring initiation of hemodialysis. Anti-myeloma therapy with VCD was commenced early during admission, and dialysis was transitioned to MCO membranes shortly thereafter. 

The patient completed 6 MCO sessions, with a marked decline in circulating free light chains. Progressive recovery of urine output was observed during the treatment course, followed by gradual improvement in renal function. He was discharged dialysis-independent with residual renal dysfunction (serum creatinine 5.5 mg/dL), which continued to improve during follow-up, reaching 1.7 mg/dL. 

### Patient 6 

A 76-year-old man with a history of IgG/κ multiple myeloma, previously treated with daratumumab-based therapy, presented with acute pulmonary edema and severe renal deterioration (serum creatinine 8.7 mg/dL). Laboratory evaluation demonstrated a marked increase in FLC (16,524 mg/L), highly suggestive of relapsed CN. Urgent hemodialysis was initiated due to volume overload and severe renal dysfunction and subsequently transitioned to MCO membranes early during treatment. Given ongoing hematologic progression, therapy was escalated to a carfilzomib-based regimen. 

The clinical course was complicated by infectious and hemorrhagic events, leading to discontinuation of MCO dialysis after 6 sessions. Nevertheless, a sustained decline in circulating free light chains was observed, accompanied by gradual recovery of urine output and renal function. Hemodialysis was discontinued during follow-up, with serum creatinine improving to 1.5 mg/dL. 

## Discussion and conclusion 

CN is a nephrological emergency in multiple myeloma in which rapid reduction of circulating FLC is central to limiting ongoing tubular injury. In this retrospective single-center case series, we describe our institutional experience implementing a standardized protocol combining contemporary anti-myeloma therapy with MCO hemodialysis in patients presenting with acute kidney injury requiring hemodialysis and presumed cast nephropathy. 

Dialysis independence at 90 days was achieved in 50% of patients, a rate comparable to those reported in a contemporary series of dialysis-dependent myeloma patients treated with modern bortezomib-based regimens [1]. Although the small sample size precludes formal comparison, these findings suggest that the combined strategy adopted in our center is aligned with current expected outcomes in this high-risk population. 

A consistent observation across most patients was the rapid decline in circulating FLC during the initial intensive MCO phase. In 4 of 6 patients, FLC reduction exceeded 80% by the end of the MCO treatment period. These findings are consistent with prior reports suggesting that MCO membranes permit effective extracorporeal clearance of circulating light chains when used alongside contemporary anti-myeloma therapy [6, 7]. 

The potential role of MCO dialysis as an extracorporeal strategy for FLC removal beyond conventional dialysis indications remains uncertain. Although the observed reduction in circulating FLC is biologically plausible and may resemble apheresis-like approaches, current evidence does not support its use in the absence of established indications for dialysis. 

Renal recovery, however, was not uniform and appeared to be influenced by multiple clinical factors. In patient 2, repeated episodes of clinical instability related to infectious complications led to interruption of the planned extracorporeal schedule, potentially limiting the cumulative effect of FLC clearance despite an initial biochemical response. Patient 4 experienced a substantial delay in initiation of chemotherapy and MCO dialysis due to clinical instability, infectious complications, and late nephrology referral, which may have allowed prolonged exposure to high FLC levels before effective intervention. Conversely, Patient 3 demonstrates that even substantial and sustained FLC reduction does not invariably translate into dialysis independence, underscoring the multifactorial nature of renal reversibility in CN. 

Taken together, these observations suggest that while rapid FLC reduction is a necessary therapeutic objective, it may not be sufficient in isolation. Timing of intervention, continuity of therapy, systemic complications, and the extent of established renal injury all likely interact to determine renal outcome. In our experience, close coordination with hematology allowed early initiation of anti-myeloma therapy, and extracorporeal strategies were implemented without delaying systemic treatment. 

Beyond individual patient outcomes, this experience has informed progressive refinement of our institutional pathway. Over time, earlier recognition of myeloma-associated acute kidney injury, more systematic FLC measurement, and more coordinated initiation of combined therapy have been incorporated into clinical practice. While the present study is not designed to evaluate temporal trends, such organizational evolution may contribute to more timely and structured management in future patients. 

This study is limited by its retrospective design, small sample size, absence of a control group, and descriptive nature, which preclude statistical inference or assessment of the independent contribution of MCO therapy. Larger prospective cohorts will be required to determine whether early and sustained extracorporeal FLC removal translates into measurable improvements in renal and patient outcomes. Changes in circulating FLC levels reflect the combined effect of extracorporeal removal and chemotherapy and do not allow isolation of the specific contribution of MCO dialysis. Additionally, all patients in this series had kappa FLC-associated disease, which may limit extrapolation of these findings to λ FLC, given their different molecular characteristics and extracorporeal clearance profiles. 

## Ethical standards 

The study was conducted according to the principles of the Declaration of Helsinki. 

## Authors’ contributions 

Ana Rita Ramos conceptualized the study, designed the institutional protocol framework, collected and analyzed clinical data, performed data interpretation, and drafted the manuscript. 

Marisa Roldão contributed to patient management, implementation of the dialysis protocol, clinical data acquisition, and critically revised the manuscript for important intellectual content. 

Rita Valério Alves contributed to patient care, data collection and interpretation, and participated in manuscript revision. 

Rachele Escoli contributed to protocol implementation, dialysis management, and manuscript revision for intellectual content. 

Paulo Santos supervised the study, contributed to study design and interpretation of findings, and critically revised the manuscript for important intellectual content. 

All authors approved the final version of the manuscript and agree to be accountable for all aspects of the work. 

## Funding 

This study received no external funding. 

## Conflict of interest 

None declared. 

**Table 1.** Patient characteristics and outcomes.

**Patient**	**Age/sex**	**Myeloma type**	**Baseline creatinine (mg/dL)**	**Baseline FLC (mg/L); κ/λ ratio**	**Regimen**	**Time to chemotherapy (days)**	**FLC at MCO start (mg/L)**	**Time to MCO (days)**	**MCO sessions**	**Maximal FLC reduction (%)**	**Creatinine at discharge (mg/dL)**	**Dialysis independence**	**Follow-up (days)**	**Final creatinine (mg/dL)**
1	77/ F	IgG/κ	5.9	κ 30000; κ/λ 3666	VCD	12	38,310	15	16	99.7	2.8	Yes	92	0.8
2	76/ F	IgA/κ	10	κ 20671 mg/L; κ/λ 5129	VCD	10	46,579	7	9	81.8	NA	No	232	NA
3	63/ M	IgG/κ	4.8	κ 1550; κ/λ 308	VCD	13	1,854	15	14	89.1	NA	No	90	NA
4	73/ M	κ	5.3	κ 2577; κ/λ 311	VCD	35	2,188	56	21	87.2	NA	No	72	NA
5	82/ M	IgA/κ	7.5	κ 4205; κ/λ 391	VCD	6	2,164	9	6	45.8	5.5	Yes	114	1.7
6	76/ M	IgG/κ	8.7	κ 16524; κ/λ 8752	KPd	6	19,315	4	6	42.1	NA	Yes	58	1.5

Baseline serum creatinine and dialysis requirement refer to values at hospital admission. Time to chemotherapy and time to MCO correspond to the number of days from hospital admission to initiation of anti-myeloma therapy and MCO dialysis, respectively. Maximal FLC reduction (%) represents the percentage decrease from baseline to the end of the MCO treatment period. Follow-up refers to the time from initiation of MCO dialysis to the last available clinical and laboratory assessment. Dialysis independence reflects recovery of kidney function at any time during follow-up, whereas final creatinine corresponds to the last available clinical assessment. FLC = free light chains; κ/λ = kappa/lambda ratio; MCO = medium cut-off; VCD = bortezomib–cyclophosphamide–dexamethasone; KPd = carfilzomib–pomalidomide–dexamethasone; NA = not applicable.
